# Gender roles and gender norms associated with psychological distress in women and men among the Dutch general population

**DOI:** 10.1177/13591053231207294

**Published:** 2023-11-06

**Authors:** Paula MC Mommersteeg, Irene van Valkengoed, Paul Lodder, Robert-Paul Juster, Nina Kupper

**Affiliations:** 1 Tilburg University, The Netherlands; 2 University of Amsterdam, The Netherlands; 3 University of Montreal, Canada

**Keywords:** femininity, gender, gender role, masculinity, psychological distress, sex

## Abstract

Women report more psychological distress than men, which may be related to both biological sex and socio-cultural gender. We tested whether associations between gender and distress differ for women and men. The cross-sectional sample consisted of 678 Dutch people (54% women). Gender roles were assessed as masculinity and femininity. A composite gender norm score was calculated by summing gendered sociodemographics. Multivariate regressions examined sex, gender, and their interaction for depressive symptoms, anxiety, and perceived stress, additionally adjusted. Women reported more psychological distress. People scoring higher on masculine gender roles, but not feminine gender roles, reported lower psychological distress. A higher gender norm score was related to more depressive symptoms and perceived stress. This association was only present in men and was explained by health-related covariates. This research shows that there is a need to further elaborate on the discrepancies between sex and gender in health psychology research to better understand individual differences.

## Introduction

While it has been suggested that mental health research should differentiate between biological sex and sociocultural gender (Heidari et al., 2016), little research to date examines the combination of sex and gender with regards to psychological distress. The terms sex and gender are often used interchangeably or remain neglected in most health science literature (Orth-Gomér and Deter, 2015); however, there is an important distinction among these inter-related yet orthogonal constructs. Sex in general refers to the biological attributes of chromosomes, gene expression, hormones, and reproductive anatomy (Clayton and Tannenbaum, 2016; Schiebinger and Hinds, 2010). In the present study, sex is operationalized as self-reported sex, with the option “woman” or “man.” Simultaneously, a person is born and raised in a gendered society and exposed to societal norms that influence their health and well-being. Gender here refers to the social and cultural norms that shape “feminine,” “masculine,” “androgynous,” and non-binary identities, expressions, and behaviors (Clayton and Tannenbaum, 2016; Schiebinger and Hinds, 2010). At the societal level, *gender norms* shape interests, expectations, and divisions of labor for women, men, girls, boys, and gender diverse people (Schiebinger and Hinds, 2010; Tadiri et al., 2021). At the individual level, gender comprises one’s *gender identity*, *gender related traits*, and *gender roles* (Clayton and Tannenbaum, 2016; Nielsen et al., 2021). In the present study one’s gender identity was not further operationalized, and neither did we have information on intersex variations.

From the perspective of a biopsychosocial model of health and disease, there is a close interaction between biological, psychological, and social factors (Wade and Halligan, 2017). In the present study we aim to examine the associations among biological sex, socially constructed gendered variables, and mental health in a community sample of women and men between 18 and 85 years. Sex differences exist for mental health conditions. For example aspects of psychological distress such as depressive symptoms, perceived stress, and anxiety are more prevalent in women than men (WHO, 2017). In particular, psychological distress is not only relevant for mental wellbeing, but also affects physical health (Mayor, 2015), such as incident cardiovascular disease (Smaardijk et al., 2019), diabetes (Mommersteeg et al., 2012), and cancer (Chida et al., 2008). It is therefore imperative to further explore sex differences in psychological distress with consideration of gendered variables, gender roles, and gender norms.

Gender roles in the present study represent gendered traits and behaviors related to the self-identification with stereotypical masculinity and femininity (Hoffman and Borders, 2001). This is manifested as distinct gendered traits that are shaped by behavioral norms imposed by traditional patriarchal and matriarchal systems more broadly (Hoffman and Borders, 2001). Feminine gender roles include profiles stereotypically considered feminine, such as being sympathetic, sensitive to others’ needs, gentle, and warm, which is summarized as “communion” thus orienting to well-being of others (Eagly et al., 2020). In contrast, masculine gender roles include profiles stereotypically considered masculine, such as being dominant, having a strong personality, acting as a leader, and making decisions easily, which is termed “agency,” or orientation toward one’s own goals (Eagly et al., 2020). Research suggests that adults who endorse masculine traits report a better mental health, whereas adults who endorse feminine traits more often reported elevated anxiety (Arcand et al., 2020; Matud et al., 2019; Mayor, 2015). At the same time, a masculine discourse (a cultural representation) is that men find it harder to admit to anxiety over health symptoms (Payne, 2014). To date, the support for associations among gender roles and psychological distress is limited (Matud et al., 2019), and comparisons with sex are lacking.

Gender roles can also reflect gender norms, defined by the unspoken rules of society, related to schools, workplaces, family, communities, and culture (Graham et al., 2017; H2020_ExpertGroup, 2020). For example, there is a dominant discourse about gender embedded within the school environment, reinforcing a stereotypical binary gender discourse (Graham et al., 2017). Gender roles influence one’s attitudes, social interactions, personality traits, and behaviors (Clayton and Tannenbaum, 2016). At a higher level surrounding the individual are gender norms that are present in all aspects of everyday life. For example, gender norms become apparent in the gender inequalities between men and women in paid work participation, earnings, pensions, hours of work, caretaking, and division of unpaid work at home (OECD, 2017).

In the Netherlands, 74% of women participate in part-time work (12–35 hours/week), versus 23% of adult men (Portegijs and van Den Brakel, 2018). At the same time, women are more likely to be engaged in caretaking and household responsibilities compared to men (60% vs 40%; Portegijs and van Den Brakel, 2018). In terms of civil status, women experience a greater loss of income when divorced or widowed compared to men (Vaart et al., 2020), and an estimated 34% of women, compared to 19% of men, are not economically independent (Brakel and Riele, 2022). A lower socioeconomic status, as indicated by income (men are more often primary earners) or education level (in older generations men are more often higher educated) is associated with more health care visits and expenditure, among others for mental health outcomes (Loef et al., 2021). Based on this, one can hypothesize that more psychological distress may be experienced by women and people with a higher gender norm score.

Gender norms are likely associated with psychological distress and mental health. To date, however, there are only a limited number of studies available on any single characteristic of sex or gender. For instance, women who work report better mental health than women who do not work, whereas caregiving is related to adverse mental health (Mayor, 2015). Informal caregiving is more prevalent in women, and this has been related to more depressive symptoms and anxiety compared to caregiving in men (Rexhaj et al., 2023).

In addition to the study of separate gender role or gender norm indicators, studies have demonstrated the possibility to *combine* gender-related aspects into a composite gender score (Pelletier et al., 2015; Smith and Koehoorn, 2016; Tadiri et al., 2021). Our goal in this study is to test a similar approach that assesses the differential associations that sex and gender exert on mental health. Specifically, we assessed psychological distress that is associated with numerous psychological conditions like depression and anxiety. In the present study, we examined the association of sex and gender (i.e. gender roles and gender norms) in association with self-reported psychological distress in the general Dutch population. Associations of gender role and gender norm with depressive symptoms, anxiety, and perceived stress were reported separately unadjusted, adjusted for sex, and with the interaction between sex and the gender variable. Furthermore, a final model included adjustment for potential covariates known to be associated with psychological distress; age and health-related variables body mass index (BMI), smoking, and exercise (Bonnet et al., 2005).

We hypothesized that masculinity was negatively, and femininity positively associated with psychological distress, independent of sex. In addition, we expected that a higher composite gender norm score was related to higher levels of psychological distress. The potential interactions of sex with gender were additionally explored using sex stratified analysis to assess whether sex modified the effect of gender on psychological distress. It can be hypothesized that women with a higher masculine gender roles as well as a lower gender norm score experienced lower psychological distress. By contrast, it can be hypothesized than men with higher feminine gender roles as well as a higher gender norm score experienced higher psychological distress.

## Materials and methods

### Participants and procedure

Data were collected in a convenience community sample of women and men between 18 and 85 years residing in the Southern provinces of the Netherlands in 2019. Quota sampling ensured inclusion of an equal number of men and women, equally dispersed over each age decade between 18 and 85. Note that this study did not include items for gender identity that would have allowed representation of cisgender, transgender, gender non-binary, and gender diverse people. In addition, we did not include items that would have identified intersex or people with variations in sexual characteristics. Participants were approached by research assistants, providing information on the study on paper and orally. Most data (97%) were collected online via Qualtrics, or via paper questionnaires. The study was approved by the ethics review board of Tilburg University (EC-2012.23), and 716 participants signed informed consent. After exclusion of incomplete data on sex, the total dataset comprised 678 people.

### Sex and gender

#### Sex

We asked people to report their sex in the survey, coded as woman (1), or man (0). In effect, this more accurately represents a measure of “sex/gender” since it does not distinguish between birth-assigned sex and gender identity. Consequently, intersex, transgender, gender non-binary, and gender diverse people may be included in our sample but cannot be identified as such.

#### Gender roles

The 12-item Dutch version of the Bem Sex Role Inventory (BSRI-12) was used to examine feminine and masculine gender traits (Bem, 1974). The 12-item short form has been validated and was extracted from the 30-item Dutch version of the questionnaire (Willemsen and Fischer, 1996) (Supplemental Table S1). Each characteristic was applied on a 7-point Likert scale from 1 (never to almost never) to 7 (always). We conducted confirmatory factor analysis (CFA), which confirmed the two-factor structure of this instrument sub-divided into masculine and feminine sub-scales (for details, please see online supplement). McDonald’s Omega (0.89 and 0.87 respectively for masculinity and femininity) suggested good internal consistency (Supplemental Table S1).

#### Gender norms

Seven sociodemographic variables were included in our composite gender norm score: civil status, education level, employment status, primary earnership, household responsibilities, and caretaking responsibilities. Variables were first recoded into gender-sensitive variables to conform with the approach developed by Smith and Koehoorn (2016). The choice of variables was pragmatic and explorative, based on an a priori literature search (Juster et al., 2016; Pelletier et al., 2015; Smith and Koehoorn, 2016; Tadiri et al., 2021) as well as the corresponding availability of these variables in the present dataset. Specifically, the recoding procedure involved examining each variable for prevalence among women and men in the current dataset, and then recoding the variables into three groups, in which a score of 0 was assigned to the category with the highest proportion of men, a score of 1 was given to the intermediate categories, and finally a score of 2 was given to the category which included the highest proportion of women.

#### Calculation of the gender norm score:

The gender norm score was computed as a sum of the seven gendered variables described above (range 0–14), allowing for two missing answers with replacement of the mean. A higher score indicated a feminine profile, and a lower score a masculine profile. This approach is in line with the pragmatic study-specific approach advocated for analyzing gender, at least at a sociodemographic level, in quantitative studies (Tadiri et al., 2021).

### Psychological distress

#### Depression

Depressive symptoms were captured with the patient health questionnaire (PHQ9). Items are rated on a Likert scale from 0 (“not at all”) to 3 (“almost every day”) (Kroenke et al., 2001). Internal consistency in the current sample was excellent (Cronbach’s alpha = 0.98).

#### Anxiety

Anxiety symptoms were measured with the Generalized Anxiety Disorder (GAD-7) scale, a valid and efficient tool to screen for generalized anxiety (Spitzer et al., 2006). The seven items are scored on a Likert scale from 0 (“not at all”) to 3 (“almost every day”) (Spitzer et al., 2006). Internal consistency of the scale was excellent (Cronbach’s alpha = 0.97).

#### Perceived stress

The 10-item version of the Perceived Stress Scale (PSS-10) was used to assess the level of perceived psychological stress over the past 4 weeks (Cohen et al., 1983). Items are scored on a Likert scale from 0 (“never”) to 4 (“all the time”). In the current sample, internal consistency was good (0.83).

### Health-related variables

Several health-related variables were assessed to be included in the models as covariates. This was explored because these variables may relate to sex and gender differences on the one hand, and adverse health-related variables have been (often bidirectionally) associated with increased psychological distress (Bonnet et al., 2005; Sarwer and Polonsky, 2016). Self-reported weight (kg) and height (m) were calculated into the continuous variable body mass index (BMI). Smoking status was coded as current smoker versus non-smoker (including former smokers), and alcohol use as user versus non-user. Endorsement of the statement: “I get enough exercise” on a five-point Likert scale, was dichotomized into being physically active (highest two) versus “moderate or insufficiently active” (lowest three) due to a skewed distribution.

### Statistical analysis

Sex-stratified descriptive characteristics are reported in Table 1. Differences between women and men were examined using Chi-square for categorical variables, and one-way ANOVAs for continuous variables. Pearson’s correlations were examined for the gender norm score and gender roles. To determine whether women and men who have a gender norm score that is more or less aligned to their sex report higher or lower psychological distress, we additionally compared these dichotomized groups using a median split in an explorative analysis.

**Table 1.** Sex stratified descriptive, gendered variables, and psychological distress.

	Women		Men		Test-value
	%/Mean	*N*/SD	%/Mean	*N*/SD	
	54%	366	46%	312	
Age [years]	48.08	16.58	48.31	17.07	0.03
Lifestyle factors					
Body Mass Index [BMI; kg/m^2^]	24.95	4.28	25.83	4.34	5.76*
Obese [BMI ⩾ 30]	13%	35	11%	31	0.23
Current smoker	14%	50	18%	55	2.00
Alcohol use	72%	262	83%	257	10.4**
Physical activity	59%	211	58%	177	0.11
Gendered items					
*Gender roles*					
Femininity [BSRI]	5.37	0.90	5.02	0.99	21.8***
Masculinity [BSRI]	4.46	1.01	4.97	1.02	39.8***
*Gender norm items*					
*Civil status*					
Single (0)	13%	45	18%	54	9.56**
With partner (1)	79%	279	78%	234	
Divorced/Widowed (2)	9%	31	4%	11	
*Education level*					
College education (0)	38%	138	47%	147	8.11*
Middle voc. training (1)	41%	148	38%	120	
High school or similar (2)	21%	78	14%	45	
*Employment*					
Fulltime (0)	19%	69	60%	187	139.7***
Not working (1)	35%	129	28%	87	
Parttime (2)	46%	166	12%	38	
*Primary earner status*					
Primary earner (0)	27%	92	70%	205	139.6***
Equal earners (1)	15%	52	15%	43	
Not primary earner (2)	58%	197	15%	43	
*Household responsibilities*					
Partner does most (0)	6%	20	45%	136	179.6***
Shared (1)	29%	105	34%	101	
I do most (>60%) (2)	65%	232	21%	63	
*Caretaking of children at home*					
Not responsible or N/A (0)	58%	197	61%	176	1.98
Shared (1)	18%	62	20%	58	
I am responsible (2)	24%	80	19%	55	
*Informal caregiving*					
Not responsible or N/A (0)	52%	178	53%	156	5.74
Shared (1)	36%	124	40%	116	
I am responsible (2)	12%	43	7%	20	
Gender norm score	7.23	2.29	4.40	1.79	310.8***
Psychological distress					
Depressive symptoms [PHQ9]	3.95	4.53	3.00	3.93	8.08**
Anxiety [GAD-7]	3.22	3.82	2.32	3.41	10.1**
Perceived Stress Score [PSS10]	11.98	6.73	10.49	5.99	8.84**

N/A: not applicable.

* *p* < 0.05. **p < 0.01. ***p < 0.001.

#### Imputation

The dataset contains missing values on some variables. The most likely mechanism is that these data are “missing at random.” No missing values were observed for age and sex, while 41 (6%) missing values were observed for the masculinity and femininity scores, and 129 (29%) for BMI, limiting the most complete dataset to 527 participants. In order to prevent bias (Donders et al., 2006) we imputed missing values using multiple imputation with five imputed datasets, calculated using all predictor and outcome variables. Multivariate analyses were reported using the five imputed datasets (each with *N* = 676). None of the main conclusions changed when examining the non-imputed findings.

#### Hierarchical regression models

Separate hierarchical linear regression analyses were run with either depressive symptoms, anxiety, or perceived stress as continuous outcomes. In the first step, either sex (women/men) or the continuous gender variables (either masculinity and femininity, or the gender norm score) were entered in the model. The analysis of sex was subsequently adjusted for covariates age, BMI, smoking, and exercise (Sex—Model 2a). In the gender analysis, the second model adjusted for sex (Model 2b). In model 3, the interaction of sex-by-gender was added. Finally, the fourth model additionally adjusted for the covariates age, BMI, smoking, and exercise. Standardized beta coefficients and the adjusted *R*^2^ of change for each consecutive model were reported. An additional sex-stratified model was explored using the continuous gender norm score.

Data were analyzed using SPSS version 24 (IBM Corp., Armonk, N.Y., USA). A *p*-value of 0.05 was considered significant. Open science framework repository was used to share data used for the present study (Mommersteeg et al., 2021).

## Results

### Descriptive statistics

Slightly more women than men participated (54% women). Quota sampling successfully ensured there were no age differences between men and women (*M*_age_ = 48.2 ± 16.8). With respect to the health behavioral variables, men had a significantly higher BMI, and reported a higher prevalence of alcohol use.

**Figure 1.**
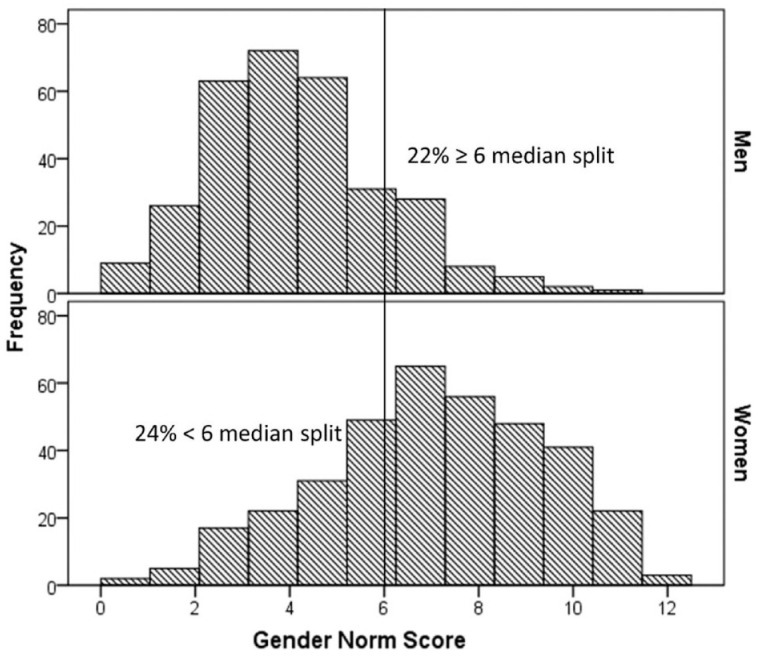
Sex stratified histogram of gender norm score distributions.

For gender roles, women reported higher levels of femininity, whereas men reported higher levels of masculinity (Table 1). Men more often reported college education and were more often the primary earner compared with women. Women more often worked part-time and had most household responsibilities compared with men. Women reported being widowed or divorced, whereas men more often reported being single. No significant differences were observed for taking care of children at home and informal caregiving. The gender norm score, depicted in Figure 1, ranged from 0 to 12, and correlated moderately negative with gender role masculinity (*r* = −0.257; *p* < 0.001), and weakly positive with gender role femininity (*r* = 0.123; *p* = 0.002). Using a median split of 6, in total 22% of men and 24% of women had a gender norm score that was not aligned with their reported sex. Additional explorative analysis with these median-split groups showed that men who had a higher gender norm score had higher levels of depressive symptoms (*F*_1,298_ = 6.7, *p* = 0.010) and perceived stress (*F*_1,297_ = 4.0, *p* = 0.046), but not anxiety symptoms (*F*_1,299_ = 0.7, *p* = 0.405). No significant differences were observed for women (Supplemental Table S2).

### Sex and gender predicting psychological distress

Women reported significantly more psychological distress compared to men, with more depressive symptoms (*β* = 0.109, *p* = 0.006), more anxiety (*β* = 0.124, *p* = 0.002), and more perceived stress (*β* = 0.121, *p* = 0.002). This explained between 1.2% and 1.6% of the variance of psychological distress, independent of the included covariates (Model 2a). In each model adjustment for covariates showed that being younger, smoking, and being less physically active was significantly associated with more psychological distress (Table 2). In contrast, a higher BMI was associated with less perceived stress but no difference in anxiety or depressive symptoms).

**Table 2.** Multivariable regression models of sex and gender variables predicting psychological distress.

	Depressive symptoms		Anxiety		Perceived stress	
	Beta	*R* ^2^_change_	Beta	*R* ^2^_change_	Beta	*R* ^2^_change_
*Sex (woman)*						
Model 1: sex	0.109**	**1.2%**	0.124**	**1.6%**	0.121**	**1.5%**
Model 2a: sex + covariates	0.119**		0.124**		0.117**	
Covariates		**13.0%**		**11.5%**		**10.5%**
Age	−0.206***		−0.228***		−0.160***	
BMI	−0.006		−0.058		−0.114*	
Smoking	0.187***		0.109**		0.139***	
Physical activity	−0.179***		−0.173***		−0.180***	
*Gender roles*						
Model 1: Masculinity	−0.218***	**4.9%**	−0.187***	**3.5%**	−0.258***	**7.2%**
Femininity	−0.021		0.013		−0.054	
Model 2b: Masculinity	−0.201***		−0.165***		−0.239***	
Femininity	−0.034		−0.004		−0.069	
Sex	0.066	0.4%	0.085*	0.7%	0.076	0.5%
Model 3: Masculinity	−0.180**		−0.143*		−0.207***	
Femininity	−0.077		−0.059		−0.088	
Sex	−0.081		−0.125		0.105	
Sex × Masculinity	−0.090	0.2%	−0.093	0.3%	−0.136	0.1%
Sex × Femininity	0.253		0.322		0.115	
Model 4: Masculinity	−0.228***		−0.190**		−0.236***	
Femininity	−0.055		−0.032		−0.062	
Sex	−0.236		−0.287		−0.012	
Sex × Masculinity	0.072		0.062		−0.027	
Sex × Femininity	0.250		0.322		0.113	
*[covariate adjusted]*		13.2%		11.7%		10.0%
*Gender norm*						
Model 1: Gender norm	0.122**	**1.5%**	0.076*	**0.6%**	0.102**	**1.0%**
Model 2b: Gender norm	0.083		0.007		0.049	
Sex	0.069	0.3%	0.122**	**1.0%**	0.093*	**0.6%**
Model 3: Gender norm	0.186*		0.057		0.175*	
Sex	0.238*		0.203		0.299**	
Sex × Gender norm	−0.250	0.4%	−0.121	0.01%	−0.305*	0.6%
Model 4: Gender norm	0.124		−0.001		0.117	
Sex	0.166		0.112		0.205	
Sex × Gender norm	−0.120		0.017		−0.168	
*[covariate adjusted]*		12.4%		11.2%		10.1%

Standardized coefficient beta, and *r*^2^ of change are reported. Bold typeface reflects significant associations.

Model 1 includes the main variable of interest (either sex, or masculine and feminine gender roles, or gender norms).

Model 2a adds the covariates age, BMI, smoking, and physical activity to the model describing sex differences. Model 2b adds sex to the model with gender. Model 3 adds the interaction between sex and gender (roles or norms). Finally model 4 includes covariates to the model 3 variables.

* *p* < 0.05. **p < 0.01. ***p < 0.001.

#### Models for gender roles

A higher score on masculine gender roles, but not femininity, was significantly related to less depressive symptoms (*β* = −0.228, *p* < 0.001), less anxiety (*β* = −0.190, *p* = 0.003), and less psychological stress (*β* = −0.236, *p* < 0.001). A higher masculinity score explained 4.9% (depressive symptoms), 3.5% (anxiety symptoms), and 7.2% (psychological stress) of the respective variance. Adding sex to the model did not affect these associations, and no significant interaction with sex was observed, suggesting that (a) substantial variance between sex and gender is shared, and (b) gender roles do not have a different effect in men and women.

#### Models for gender norms

Having a higher gender norm score was related to more depressive symptoms (*β* = 0.12, *p* = 0.002), anxiety (*β* = 0.10, *p* = 0.049), and psychological stress (*β* = 0.10, *p* = 0.009). However, the explained variance was low, between 0.6% and 1.5%. Depressive symptoms were significantly higher in women, and associated with a higher gender norm, though there was no significant sex-by gender interaction. These associations became nonsignificant after further adjustment for covariates. Similarly, women reported significantly more anxiety, but in the model with both sex and gender, the gender norm was no longer associated with anxiety, nor was there a significant sex-by-gender norm interaction. Perceived stress was higher in women and associated with a higher gender norm score, and, before adjustment for covariates, there was a significant sex-by-gender interaction. We additionally explored sex-stratified associations between the continuous gender norms and psychological distress to get an idea of the sex-by-gender interactions. Supplemental Table S3 shows that only in men having a higher gender norm was significantly associated with more depressive symptoms (*β* = 0.145, *p* = 0.014), and perceived stress (*β* = 0.135, *p* = 0.019), explaining about 2% of the variance.

## Discussion

The current study in the general Dutch population found associations for both sex and gender with psychological distress. Notwithstanding, the magnitudes of these associations and explained variance was rather low. To reiterate, women reported more psychological distress than men, and in an unadjusted model a higher gender norm score was also associated with more psychological distress. Sex and gender shared a substantial part of the (small) explained variance in individual differences in psychological distress. Men and women scoring higher on masculine gender roles reported lower psychological distress, independent of sex and health-related covariates. By contrast, feminine gender role was not associated with any of the outcomes. For gender roles and most analyses of the gender norms score, the effect of gender was not modified by sex. However, only in men was having a high gender norm score, associated with more depressive symptoms and perceived stress. These associations were explained by the health-related covariates, showing that being younger, smoking, and being less physically active was significantly associated with more psychological distress.

Women showing higher levels of depressive symptoms, anxiety, and perceived stress than men is well established, and in line with general findings on a national and global level (Statline CBS, 2011; Verweij and Herten, 2013; WHO, 2017). Our finding that masculine gender roles were related to lower psychological distress in both men and women is in line with previous studies (Arcand et al., 2020; Mayor, 2015). However, no significant associations between feminine gender roles and with psychological distress were observed, which contrasts previous findings (Arcand et al., 2020). The study of Arcand et al. (2020) stratified their findings for students (22 years) and workers (35 years). In the present study, we did not create this stratification as people from across the entire adult lifespan were represented. We postulate that self-identification with gendered stereotypes such as feminine gender roles and gender norm changes across generations and life stages. Moreover, these may be further affected by civil status, taking care of children, and educational/occupational status. While it was beyond the scope of the present paper to examine age groups in more detail, future research would do well to explore age, sex, gender, and other intersecting factors in this regard.

Masculinity gender role refers to how people identify with regards to profiles traditionally associated with behavioral expectations for men. This masculine gender role can be seen as a combination of the personality characteristics that represent “agency” and “instrumentality” (Mayor, 2015). Agency is defined as the capacity of individuals to act independently and to make their own free choices (Eagly et al., 2020), and instrumentality is described as “an individual’s striving for independence, mastery, task accomplishment, and self-assertiveness” (Stake, 1997). The current findings as well as previous findings suggest that these aspects of masculine gender roles may be protective against experiencing psychological distress. At the same time, using masculinity and femininity based on the Bem’s 50 year old instrument is now regarded as being outdated and not representative, since the concepts were developed in mostly higher educated, white, and undergraduate students (Nielsen et al., 2021). Notwithstanding this limitation, the present study confirms that masculinity has explanatory value for psychological distress in addition to sex. The absence of a significant interaction with sex suggests these personality constructs may be equally relevant for women and men.

Recently, authors have advocated for the construction of a composite gender norm score (Nielsen et al., 2021; Tadiri et al., 2021) in order to capture socio-culturally gendered behavioral patterns that might also explain mental health differences. While several studies have constructed such gender norm scores, for example, based on a list of gendered demographic and personal characteristics that varies in content and calculation per study (Ballering et al., 2020; Mayor, 2015; Pelletier et al., 2015; Smith and Koehoorn, 2016), none of these previous studies have examined the association of such a score with mental well-being. In the current paper, we constructed a gender norm score based on differences in compliance with norms observed in Dutch society between men and women, and consistent with a global division of labor and caretaking report (OECD, 2017).

Before adjustment for sex and covariates, a higher gender norm score was significantly associated with more depressive symptoms, anxiety, and perceived stress, explaining between 0.6% and 1.5% of the variance in these variables. With cautious interest, the interaction with sex, and sex-stratified explorative findings show that in men, but not women, a higher gender norm score, is associated with higher depressive symptoms, and perceived stress. This may be in line with existing evidence showing that gender nonconformity increases mental distress in adults (Zentner and von Aufsess, 2022). Our results, obtained in a country with relatively fair gender equality (#38 out of 153 countries on the gender equality ranking list) (World Economic Forum, 2019), could suggest that not meeting a gender norm is more detrimental for mental health in men than in women. While we cannot confirm this association, our work does provide creative ways from which to measure gender norms at the more societal-level than simply individual-level gender roles that we note are flawed.

There are several considerations regarding the individual variables that went into the gender norm score that warrant discussion. Regarding civil status, for example, men reported to be single more often, whereas women typically reported to be widowed or divorced more often, which is related to a larger loss of income for women (Portegijs and van Den Brakel, 2018). It may be that men are more likely to identify themselves as single, even when widowed or divorced. On the other hand, women tend to suffer more chronic consequences of divorce, including poverty and single parenthood (Leopold, 2018; Portegijs and van Den Brakel, 2018). Importantly, not being in a steady relationship is associated with a higher mortality risk, compared to married people, particularly in divorced/separated men (Wang et al., 2020), which makes civil status an important health predictor in its own right. In the current study, women also typically reported a lower educational level than men.

This contrasts findings in the general population whereby women are nowadays more often higher educated than men (OECD, 2017). This observation, however, concerns the just graduated, and not the entire Dutch population. As our samples spanned the adult age range, this might have affected the gender distribution for educational level. In addition, having a lower gender norm score in women (i.e. not being a homemaker, having a skilled profession) has previously been associated with improved mental wellbeing (Matud et al., 2019). By contrast in the current study, the gender norm score was generally unrelated to psychological distress in women. Masculinity in women, in accordance with our own results, could serve as an explanation for the findings.

We a priori expected our health-related covariates to show both gender differences and be associated with increased psychological distress (Bonnet et al., 2005). Adverse health behaviors like smoking, and lack of physical exercise were associated with psychological distress in the present study, explaining between 11% and 13% of the variance. Adding these covariates to the gender models increased the association of masculinity with psychological distress, which is suggestive of some unmasking of variance. It will be important for future research to explore compounding effects of gender variables in relation to gendered health-related behaviors.

Several limitations and strengths of the current study are worth mentioning. People self-reported their sex as either men or women, which limits our information on self-identification within people with an intersex variation, people identifying on a broader gender spectrum, as well as women and men born with a different sex. A recent large-scale cohort study in the Netherlands showed that the prevalence of intersex variations, based on genetics, is less than 1%. Results also showed that intersex statistically did not affect the constructed gender index measure in that study (Ballering et al., 2020). Not knowing about intersex variations will likely not have affected the evaluation of our gender index statistically significantly. However, ideally, we would have also asked people about their (known) intersex variations and gender identity, including the option “Prefer not to state” (Nielsen et al., 2021).

Our findings are reported cross-sectionally which precludes any causal effects. We did not aim to create nor confirm specific theoretical positions related to gender research, but chose a pragmatic approach for a data-driven quantitative approach. Despite this, many researchers do not recommend using Bem’s instrument despite our finding of sex differences consistent with Bem’s original formulation even today. This is complimented by our composite approach to assessing gender norms; however, more modern instruments, items, and approaches would have enhanced our methodologies. In the present study, gender was not identified according to an intersectional approach, which acknowledges people along multiple dimensions that include ethnicity, age, socioeconomic status, (dis)ability, and sexuality (Hankivsky, 2014). Sex-related prejudice and stereotyping may influence gender roles and norms, which would be subject for further research.

Quota sampling strengthened the generalizability of the results to the general Dutch population. Still, the Dutch population may not represent gender differences in other countries, given that, for example, parttime work for both women and men is more prevalent in the Netherlands, both with positive (a general high happiness score) and negative (less likely to be economically independent) sides, which could affect psychological distress in either direction (Brakel and Riele, 2022; Loef et al., 2021). Some of these findings may be country specific, which comes from a long history with a delay in economic independence and unequal opportunities for gaining positions of power for women in the Netherlands (Bosch, 2010).

In calculating the gender norm sum score, we chose to code “not applicable” answer to both caregiving items as “0.” While on one hand this enables these items to contribute to the total gender score, it introduces a bias toward “men” since men were coded as zero. Our rationale for this choice was that when care is deemed not applicable, it might also mean there is no responsibility. While construction of an index or composite score is justified when it covers a system, which is the case for examining gender domains (Tadiri et al., 2021), the field of study could benefit from examining the gender norms separately. This could provide more insight into socio-culturally gendered behavioral patterns, although it would involve collecting data from a much larger sample.

## Conclusion

Women reported more psychological distress than men, and a higher masculinity gender role is associated with lower psychological distress in both women and men. Sex and gender shared a substantial part of the (small) yet significant explained variance in individual differences in psychological distress. Interestingly, the effect of gender was mostly *not* modified by sex. Men with a higher gender norm score experienced more depressive symptoms and perceived stress, which co-occurred with poor health behaviors. These findings show the added value of examining gender in addition to sex and indicate a need to further elaborate on the discrepancies between sex and gender in psychological and health research.

## Supplemental Material

sj-pdf-1-hpq-10.1177_13591053231207294 – Supplemental material for Gender roles and gender norms associated with psychological distress in women and men among the Dutch general population Supplemental material, sj-pdf-1-hpq-10.1177_13591053231207294 for Gender roles and gender norms associated with psychological distress in women and men among the Dutch general population by Paula MC Mommersteeg, Irene van Valkengoed, Paul Lodder, Robert-Paul Juster and Nina Kupper in Journal of Health Psychology
