# Supplementary material for: Gender roles and gender norms associated with psychological distress in women and men among the Dutch general population
Source: J Health Psychol. 2023 Nov 6;29(8):797–810. doi: 10.1177/13591053231207294 (PMC11292987; doi:10.1177/13591053231207294)
Supplement: sj-pdf-1-hpq-10.1177_13591053231207294 – Supplemental material for Gender roles and gender norms associated with psychological distress in women and men among the Dutch general population [file sj-pdf-1-hpq-10.1177_13591053231207294.pdf]

Online supplement – Methods section

Table S1: Original and Dutch items of the BSRI 12 item version.

Bem items English	Dutch items
Makes decision easily. M.	BSRI 1: Ik neem gemakkelijk beslissingen.
Affectionate. F.	BSRI 2: Ik ben hartelijk.
Leadership abilities. M.	BSRI 3: Ik heb leiderschapskwaliteiten.
Strong personality. M.	BSRI 4: Ik ben een sterke persoonlijkheid.
Gentle. F.	BSRI 5: Ik ben zachtmoedig.
Sensitive to other's need. F.	BSRI 6: Ik ben gevoelig voor de behoeften van anderen.
Tender. F.	BSRI 7: Ik ben teder.
Warm. F.	BSRI 8: Ik ben warmhartig.
Defend own beliefs. M.	BSRI 9: Ik verdedig mijn eigen opvattingen.
Acts as a leader. M.	BSRI 10: Ik gedraag mij als een leider.
Sympathetic. F.	BSRI 11: Ik ben meevoelend.
Dominant. M.	BSRI 12: Ik ben dominant.

BSRI = Bem sex role inventory; F = feminine item; M = masculine item.

Confirmatory factor analysis of Bem Sex role inventory

Model fit indicators showed that the 2-factor model including a masculinity component and a femininity component fit the data better than a 1-factor model. However, in general, the model still showed a poor fit. After inspection of the CFA results, we decided to run an additional model, removing three items (1 'makes decisions easily', 9 'defend own beliefs', 12 'dominant') from the masculinity component. As is visible in Figure 1 below, these items showed a lower association with the masculinity factor (.54-.58). Removing the three items improved fit, as can be seen in the table below (Table 1: model fitting results). However, for comparability reasons, we kept the three masculinity items in, as previous studies have also used the complete scale. Future research is encouraged to further examine the factor structure of the Bem sex role inventory, and to improve the masculinity subscale.

Table 1: CFA model fitting results

	**Robust Chi square**	**df**	**χ^2/df**	**TLI**	**RMSEA**
**1-factor model**	3429.757	54	63.51	.546	.317
**2-factor model**	1088.847	53	20.54	.858	.177
2-factor model w/o items 1, 9, 12	368.299	26	14.17	.941	.145

TLI: Tucker-Lewis index; a score of .95 or higher indicates a good model fit.
RMSEA: Root Mean square of approximation; a value of .06 or less indicates a good model fit.

Multiple fit indices were calculated, including χ^2/df ratio, Tucker-Lewis index (TLI), and root mean square error of approximation (RMSEA). Latent factors were defined to have a mean of 0 and a variance of 1 (i.e., standardized), allowing free estimation of all factor loadings. Residuals were uncorrelated, and missing values were handled ("pairwise" option).

Figure 1. Factor structure of the 12-item Bem Sex role inventory (top), and the best fitting model, including 9 items (bottom)

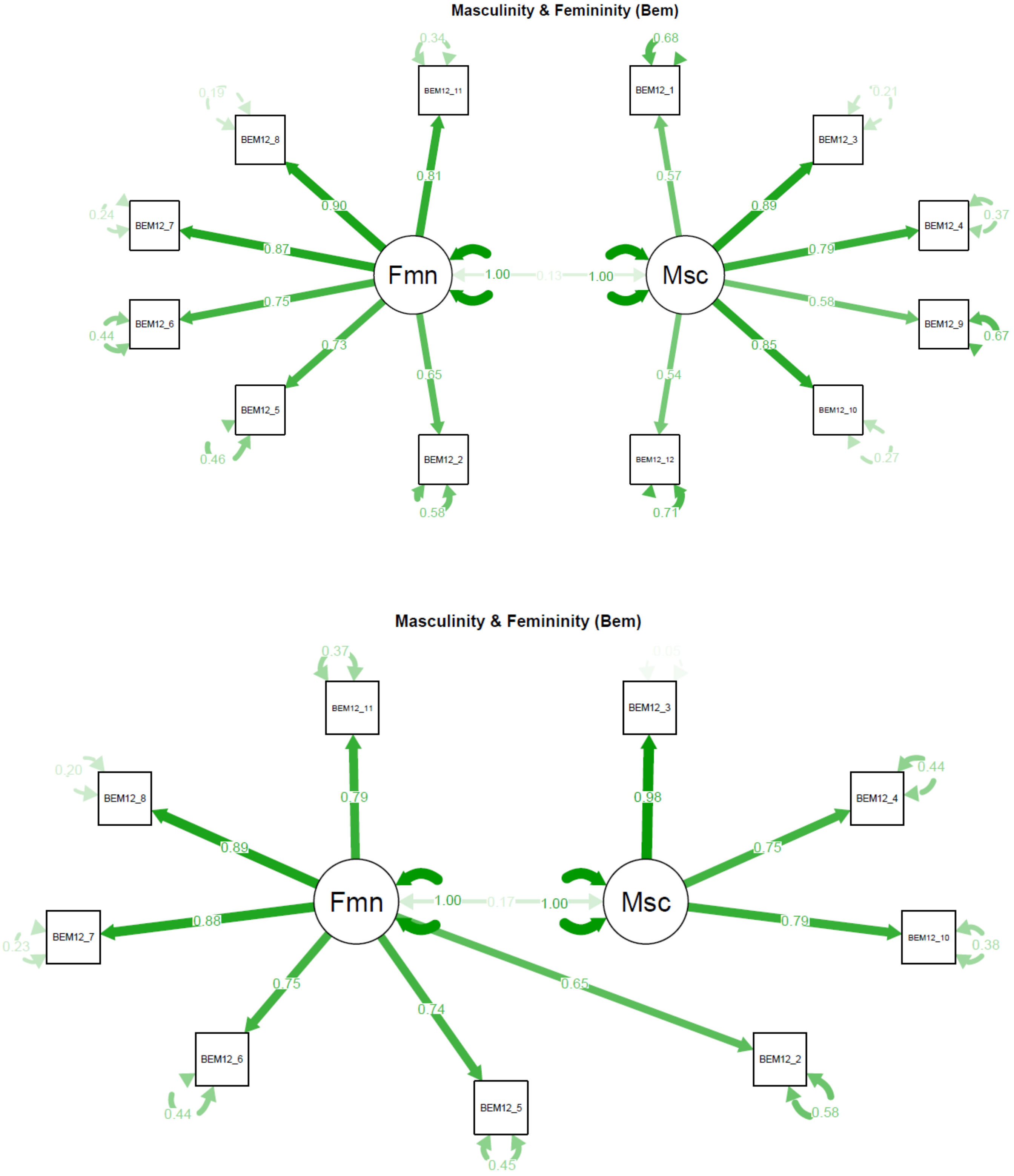

Online supplement – Results section

Supplemental Table S2.

Explorative analysis of differences in depressive symptoms, anxiety, and perceived stress in median-split group of gender norm conform a propensity with their sex or not in women and men.

	Gender norm conform sex		Gender norm other than sex				
	Mean	SD	Mean	SD	F-value	Degrees of freedom	p-value
**Women:**							
Depressive symptoms [PHQ9]	4.03	4.64	3.76	4.22	0.22	1; 350	0.641
Anxiety [GAD7]	3.19	3.82	3.38	3.89	0.17	1; 349	0.684
Perceived stress [PSS]	12.04	6.82	11.83	6.49	0.07	1; 349	0.797
**Men:**							
Depressive symptoms [PHQ9]	2.69	3.72	4.12	4.50	6.72	1; 298	0.010
Anxiety [GAD7]	2.24	3.43	2.64	3.38	0.70	1; 299	0.405
Perceived stress [PSS]	10.13	5.83	11.83	6.45	4.00	1; 297	0.046

Using a median-split of 6, in total 24% (N=85) of women and 22% (N=65) of men had a gender norm score not aligned with their sex.

Supplemental Table S3.

Explorative sex-stratified associations of the gender norm score with psychological distress.

	Depressive symptoms [PHQ9]		Anxiety [GAD7]		Perceived Stress [PSS]	
	Beta	R^2_{change}	Beta	R^2_{change}	Beta	R^2_{change}
**Women:**						
Gender norm, unadjusted	0.025	0.1%	-0.017	<0.1%	-0.015	<0.1%
Gender norm, adjusted	0.036	15.2%	0.004	14.1%	0.011	14.8%
**Men:**						
Gender norm, unadjusted	**0.145***	**2.1%**	0.044	0.2%	**0.135***	**1.8%**
Gender norm, adjusted	0.089	12.9%	-0.002	10.6%	0.097	8.2%

A higher gender norm score indicates a feminine profile. R^2_{change} represents the percentage variance of change in the total model before adjustment (unadjusted) and after adjustment for covariates.

*p<.05; imputed models are shown; adjusted for age, BMI, smoking, and physical activity
